# Filial Maturity and Caregiving to Aging Parents

**DOI:** 10.3390/geriatrics9010017

**Published:** 2024-02-01

**Authors:** Diana Morais, Carla Faria, Lia Fernandes

**Affiliations:** 1 Instituto Politécnico de Beja, 7800-295 Beja, Portugal; 2 Observatório das Dinâmicas de Envelhecimento do Alentejo—ODEA, 7800-295 Beja, Portugal; 3 CINTESIS-Centro de Investigação em Tecnologias e Serviços de Saúde, 4200-450 Porto, Portugal; cfaria@ese.ipvc.pt (C.F.); lfernandes@med.up.pt (L.F.); 4 Instituto Politécnico de Viana do Castelo, 4900-347 Viana do Castelo, Portugal; 5 Faculdade de Medicina, Universidade do Porto, 4050-313 Porto, Portugal

**Keywords:** filial caregiving, filial maturity, attachment, aging parents, middle-aged children

## Abstract

The aging of parents results in changes in the filial relationship. The increasing vulnerability of parents leads adult children to realize that they have individual needs and cannot fully function as sources of security and protection, as they did before. Simultaneously, the evidence of losses and disability imposes the need for care, which tends to be assumed by adult children. Therefore, there is a progressive change in the volume of support exchanges between parents and children, with more support from adult children to parents. The way adult children adapt to these transitions is influenced by several internal and relational factors. Filial maturity has been associated with filial caregiving towards aging parents. The concept of filial maturity describes a developmental stage in which the adult child overcomes the filial crisis, realizing and accepting that the parent also needs support and comfort and starting to relate to him/her beyond the strictly parental role. Thus, this study aims to explore the role of attachment and mental representation of caregiving in filial maturity. A total of 304 children aged between 35 and 64 years old participated in this study, with at least one of the living parents aged 65 years or older, not institutionalized. Attachment was assessed with the Adult Attachment Scale, mental representation of caregiving with the Mental Representations of Caregiving Scale and filial maturity with the Filial Maturity Measure. The results suggest that attachment, mental representation of caregiving and the interaction between the two explain 24.5% (*p* < 0.01) of variability in Comprehending and 11.1% (*p* < 0.05) of variability in Distance, two dimensions of filial maturity. These findings suggest that it is important to consider mental representation of caregiving and attachment when adult children must adapt to changes in the filial relationship and to the need to care for parents.

## 1. Introduction

The demographic, social and economic transformations that we have been witnessing since the second half of the 20th century—such as greater longevity, the postponement of parenthood and the decrease in birth rates—have led to profound changes in family structure [1]. These changes become particularly visible in middle age when the empty nest gives way to the full nest and middle-aged adults are divided between the appeals of the ascending (own parents) and descending (children) generations [2,3].

All these transformations, associated with the changes that occur, both in adulthood and with aging, have a profound impact on the nature and dynamics of the relationship between middle-aged children and aging parents [1].

### 1.1. Filial Relationships and Filial Caregiving in Middle Age

In filial relationships—relationships that are established between adult children and aging parents—the dynamic between caregiving and care-receiving throughout life develops in ways that distinguish them from other relationships. They combine the relationship’s previous progress with specifics emerging in the current developmental and family period [1,4]. One of its key aspects is the process of differentiation and individualization of adult children in relation to their parents, which begins in adolescence [5,6].

Family relationships in adult life evolve towards reciprocity, hierarchical horizontality and mutuality [7]. In this sense, the older generation abandons the authoritarian position, the younger generation starts looking at parents beyond their parental role and both actors seek to balance two roles—being dependent and being dependable [8]. However, this balance can be blurred by the increase in fragility and dependence, which often accompanies the greater longevity of the parents and which imposes the demand for filial caregiving [9].

In the transition to greater dependence of parents, adult children experience mixed, ambivalent and contradictory emotions: on the one hand, they want to be present and available, but on the other, new tasks conflict with existing routines and roles, and they may have doubts regarding how to care without taking control of the relationship [10,11,12]. This ambivalence is also manifested in parents who want both independence and connection to their adult children [13]. In this adaptation, attachment, mental representation of caregiving and filial maturity can be resources or constraints to deal with caregiving for aging parents.

### 1.2. Filial Maturity

The concept of filial maturity was introduced by Blenker in 1965 [14] to describe the change that occurs in the nature and quality of the relationship between parents and adult children. She proposed a new developmental stage beyond genital maturity and introduced the concept of filial maturity to characterize the successful transition from that to old age.

According to Blenkner [14], most individuals around 40/50 years of age experience a filial crisis, when they are confronted with the aging and increasing vulnerability of the parents and with the possibility that they cease to function as sources of support as they grow older and need the support and comfort of their children. In this way, adult children are impelled to respond to the needs of their parents—when successful, they reach filial maturity. This means they abandon the rebelliousness and desire for emancipation and separation typical of adolescence and youth. They establish a relationship with their parents beyond the limits of the filial role, seeing them in a more objective way, which encompasses not only their parental role but also their individual role and history: their own pre-existing motivations and needs. This enables adult children to take care of parents based on their actual needs, respecting their individuality, instead of infantilizing care.

After Blenker, Nydegger [15] reformulated the concept of filial maturity, distinguishing in it two dimensions: Distancing and Comprehending. Distancing encompasses the separation of the adult child from the parents and the formation of a new life as an adult, as well as the objective perception that the parent is a person with limitations. Comprehending occurs after Distancing and refers to the ability to see parents as individuals with a life history that exists independently of the parent–child relationship.

Regarding the role of filial maturity in filial care, research is scarce but existing studies show that higher levels of filial maturity are associated with less burden [16], greater ability to deal with parent caregiving and a lower tendency to opt for institutionalization without considering other options [17].

### 1.3. Attachment and Caregiving

Attachment refers to a strong and lasting emotional bond built in childhood [18]. It is based in the dynamics between caregiving and care-receiving established between mother and young child, through which the individual obtains feelings of comfort and security to deal with the demands and adversities of life.

Different experiences with the attachment figure lead to different attachment patterns or styles, expressing the individual’s beliefs about himself as someone deserving of care and affection, and about others as available and trustworthy people. These beliefs, called Internal Working Models (IWMs), are the mechanism through which early attachment influences care-seeking and caregiving behaviors, as they provide information about how the individual should act and how others will respond in other social contexts [19]. Thus, experiences of sensitivity and responsiveness with the mother lead to a secure attachment. Experiences of rejection lead to an avoidant attachment. Experiences of inconsistency and ambivalence lead to an anxious attachment.

Caregiving, according to attachment theory, aims to reduce the suffering of other people, protect them from danger and encourage their growth and development [20,21,22]. It also has a representational component with its developmental roots in the IWM of attachment relationships [23,24]. The caregiving IWMs include the model of the self as a caregiver—the extent to which the individual sees himself with proper skills and abilities to care—and the model of others who need help—the extent to which the individual considers that others deserve help [25].

To provide care, the person must have reached a certain degree of security [26]. Only then can they perceive others not only as sources of security, but also as human beings who need and deserve comfort and support [24]. Dealing with the suffering of another person can evoke two types of reactions that are very much influenced by the attachment style. Thus, secure individuals tend to show empathic compassion, focusing on the other person’s needs or suffering and trying to relieve stress for the benefit of the person who is suffering [21,27,28,29,30]. Insecure individuals tend to react with personal stress, focusing on their own discomfort, which can be alleviated by either ignoring and abandoning the situation, or helping if help is the best way to reduce the caregiver’s own discomfort [21,25,29,31].

### 1.4. Study Aim

Research has shown the relevance of attachment, mental representation of caregiving and filial maturity for understanding midlife filial caregiving. However, the way in which these variables are articulated has been analyzed little. Thus, the aim of this study is to examine the role of attachment and mental representation of caregiving in filial maturity.

## 2. Materials and Method

### 2.1. Study Design

The study employed a cross-sectional design. 

### 2.2. Participants

Participants were selected through a convenience sampling procedure. The following inclusion criteria were established: (a) age between 35 and 64, (b) had at least one living parent aged 65 or older and (c) the parent was not institutionalized.

Participants were recruited from health and social care services who were supporting the aging parents. 

Directors of the social care services approved the study and gave access permission to personal information of the adult children whose parents attended the services. Health services required the study to undergo prior review by an accredited Medical Research and Ethics Committee. 

Participants were then contacted either in person or via telephone to take part in the study. After acceptance, data collection protocols were applied directly and in person by researchers.

The application of the protocols was preceded by the guarantee of confidentiality of the data provided and informed consent, both by the participant and the researchers.

### 2.3. Variables and Instruments

Attachment: Portuguese version of the *Adult Attachment Scale* [32,33] evaluates 3 attachment styles (secure, anxious, avoidant) across 18 items rated in a 5-point *Likert* scale. Secure attachment style includes individuals who feel comfortable with intimacy, can trust others and are not afraid to be abandoned; avoidant style encompasses those who do not feel comfortable with proximity towards others, do not trust them but are not afraid of being abandoned; anxious style refers to those who feel uncomfortable with proximity towards others, do not trust them and are particularly afraid of being abandoned. The *Cronbach alpha* was 0.81, the *Spearman–Brown* coefficient was 0.84 and the *split-half* correlation coefficient was 0.83.

Mental Representation of Caregiving (MRC): Portuguese version of the *Mental Representation of Caregiving Scale* [25,34] has 27 items rated in a 7-point *Likert* scale. Items are organized into 4 factors: (1) Perceived ability to provide effective help (MRC-1); (2) Perceived ability to recognize others’ needs (MRC-2); (3) Egoistic motives for helping (MRC-3); (4) Appraisal of others as worthy of help (MRC-4). The *Cronbach alpha* ranged between 0.70 and 0.80 and all items significantly correlated with the total score of the factor they belong to.

Filial Maturity: Portuguese version of the Filial Maturity Measure [35,36] has 9 items rated in a 6-point *Likert* scale. Items are organized into two subscales: (1) Comprehending, which assesses the ability to establish an intimate, understanding and mutually supportive relationship with the parents, (2) Distancing, which refers to the children’s awareness of the parents’ faults and limitations. The *Cronbach alpha* was 0.84 for Comprehending and 0.58 for Distancing.

Parent’s Functional Status: was assessed in two domains: basic activities of daily living (BADL) and instrumental activities of daily living (IADL). Functionality in BADL was assessed by the Portuguese version of Barthel Index [37,38], which encompasses 10 BADLs—feeding, bathing, grooming, dressing, bowel control, bladder control, toilet use, transfers (bed to chair and back), mobility (on level surfaces) and stairs. Scores range from 0 to 100, with higher scores indicating greater independence. The Portuguese version of Barthel Index showed high reliability with a Cronbach’s alpha of 0.89 and an item-total correlation raging from 0.53 to 0.85. Functionality in IADL was assessed with the Portuguese version of Lawton Index [38,39], which includes eight IADLs—preparing food, housekeeping, doing laundry, shopping, using the telephone, using transportation, handling finances, and handling medications. The Portuguese version of Lawton Index shows a Cronbach’s alpha of 0.92 and an item-total correlation raging from 0.75 to 0.86. 

### 2.4. Data Analysis Procedures

Data were analyzed using the SPSS V24 statistical software. The assumptions underlying the use of parametric tests were met. The T-Test for independent samples and the Chi-Square Test were used. To explore the impact of attachment and mental representation of caregiving on the two subscales of filial maturity, hierarchical multiple regression models were used.

First, the known predictors from previous investigation were inserted, in order of importance, in the explanation of the outcome, and then the new predictors were inserted, based on theoretical importance, through the Enter and Forward methods. Predictor variables were entered in seven steps: (1) sociodemographic variables—gender, age, years of education, marital status, professional status, number of children, number of siblings (Enter); (2) attachment—secure vs. insecure (Enter); (3) the four factors of MRCS (Enter); (4) interaction between attachment and the four factors of MRCS (Forward); (5) cohabitation with the parent (Forward); (6) parent’s functionality in basic activities of daily living—independent versus dependent (Forward); (7) parent’s functionality in instrumental activities of daily living—independent versus dependent (Forward). Cases whose standardized residuals had values greater than 3 were excluded. There was an absence of multicollinearity (absence of a perfect linear relationship) between the predictors, through the Variance Inflation Factor (VIF) values, which were shown to be lower than 4, and through the Tolerance values, which were shown to be above 0.10.

## 3. Results

### 3.1. Sociodemographic Characteristics of Participants

Results for sociodemographic characterization of participants are presented in Table 1.

A total of 304 adults with a mean age of 49.17 years (SD = 7.821) participated in the study. Participants were mostly women (68.10%) with a mean age of 48.56 years (SD = 7.79) and with a higher level of education than men. Men (31.90% of the sample) had a mean age of 50.48 years (SD = 7.75). Most of the participants were professionally active (77.00%), married or living together (71.70%), averaging one child (SD = 0.92). On average, the participants lived 18.54 km away from their parents.

### 3.2. Characterization of Participants Regarding Attachment, Mental Representation of Caregiving and Filial Maturity

Most participants had a secure attachment style (56.6%) and 43.4% had insecure attachment (35.2% anxious attachment and 8.2% avoidant attachment; see Table 2).

Regarding MRC, women scored significantly higher than men on factors MRC-1 (*t* (302) = −3.80, *p* < 0.001) and MRC-2 (*t* (302) = −5.24; *p* < 0.001), which means that they perceive themselves as more able and available to care and as more able of identifying and recognizing the needs of other people. Men had higher scores on the MRC-3 factor (*t* (302) = 2.98, *p* < 0.01) (see Table 3), which means that they have more selfish motivations for caregiving (see Table 3).

Results for filial maturity indicated that, although women had higher levels of Comprehending and Distancing, the difference in relation to men is only significant for Comprehending (*t* (302) = −2.87, *p* < 0.01) (see Table 3).

### 3.3. Multivariate Analyses for Filial Maturity

Table 4 and Table 5 present results from the hierarchical regressions for Comprehending and Distancing. Regarding Comprehending, the Final Model of the regression explains 24.6% (*R*^2^) of variance in Comprehending (see Table 4). The block of variables that contributes most to the explained variance in Comprehending is the one that encompasses the MRC factors (∆R2 = 0.160; *p* = 0.000). In the Final Model (see Table 5), the significant predictors are age, the MRC-1 and MRC-4 factors, and the interaction between attachment and the MRC-2 factor (Attachment X MRC-2). Attachment and the MRC-2 factor are only significant in the Final Model when the block of variables relating to the interaction between attachment and MRC is inserted. This means that lower age, perception of more capacity and availability to provide care, more evaluation of others as deserving of help and the combined effect of attachment and mental representation of caregiving are associated with greater ability to establish relationships with parents based on intimacy, understanding and mutual support. The interaction between attachment and MRC-2 factor suggests that, in secure children, a greater ability to recognize the needs of others means a greater Comprehending dimension. Conversely, in insecure individuals, the same ability is related to less Comprehension.

Findings for Distancing showed that the Final Model of the regression explains 11.1% (*R*^2^) of variance in Distancing. The significant predictors of Distancing are attachment (∆*R*^2^ = 0.038; *p* = 0.001), the interaction between attachment and MRC-1 factor (∆*R*^2^ = 0.033; *p* = 0.001) and the interaction between attachment and MRC-4 factor (∆*R*^2^ = 0.014; *p* = 0.033). The Final Model (see Table 5) shows that the significant predictors of Distancing are attachment, the interaction between attachment and MRC-1 factor (Attachment X MRC-1), and the interaction between attachment and the MRC-4 factor (Attachment X MRC-4). The contribution of attachment is significant since it is inserted into the model, suggesting that insecure attachment is related to more Distancing. The interaction between attachment and MRC-1 factor suggests that, in secure attached children, Distancing increases when the ability and availability to provide care also increases. Conversely, in insecure attached children, Distancing decreases when the ability and availability to provide care increases. The interaction between attachment and factor 4 suggests that, in children with Secure Attachment, Distancing increases when factor 4 increases, that is, the more they evaluate others as deserving of care. On the other hand, in children with insecure attachment, Distancing is relatively constant, regardless of the value of factor 4, which indicates that there is no relationship between the two variables in insecurely attached children.

## 4. Discussion

Adult children participating in this study show different mental representations of caregiving, depending on gender: women consider themselves more capable of providing adequate and effective care to a person who needs help and are more available to get involved in that care, more able to recognize and identify other people’s needs, their requests for help and the way they feel and have less selfish motivations to care, caring altruistically, not expecting to obtain advantages, benefits or to avoid negative consequences. In fact, it is common for women to have more experience in caring, as they are more frequently faced with situations in which responsibility for caring is required of them: they tend to be the main caregivers for their children in the first months of their lives, from other family members when they become ill and from older family members [40,41,42]. However, in this study, women are more likely to be single and unemployed unlike men who are older, more likely to be married, more likely to be employed, and farther from their parents’ homes. It may happen that the female participant population may have relatively more favorable conditions for caregiving than men. 

Regarding filial maturity, Comprehending is higher in daughters than in sons. Several studies that analyze the association between the quality of the filial relationship and gender show that relationships between mothers and daughters are closer emotionally [43]. Not only do daughters feel closer to their mothers and turn to them to confide in them [4,44], but mothers also feel more positive affection towards their daughters, sharing more intimate and emotional information with them and they turn to them for comfort [4,45]. This emotional closeness between mothers and daughters can encourage the sharing of experiences between them and thus reduce conflicts [46,47,48,49]. In the context of these closer relationships, individuals are likely to be better able to understand each other [15,35].

Regression analyses show that filial maturity is essentially explained by attachment and mental representation of caregiving. These results highlight the relevance of the representational dimension of caregiving and of the attachment relationship in the development of filial maturity.

Thus, in terms of Comprehending, the interaction between attachment and MRC-2 factor suggests that, in secure attached children, greater ability to recognize the needs of others increases this dimension, while in insecure children, it decreases it. In secure children, this association makes sense, because it is the perception of signs of discomfort and suffering in others that activates the caregiving system and, consequently, the actions of approaching and relieving the discomfort [25]. At the Distancing level, the interaction between attachment and MRC-1 factor and the interaction between attachment and MRC-4 factor suggest that, as secure individuals see themselves as more capable of caring and see others as deserving of help, they also seem better able to recognize parental flaws and limitations. It should be noted that recognizing parental limitations is not a negative aspect, but a necessary condition for developing filial maturity [14,15]. Thus, these results seem to suggest that, in secure children, the development of positive representations of themselves as caregivers and of others as needing care seems to be accompanied by a more realistic and objective perspective on the parents, which allows them to integrate their weaknesses and limitations (Distancing), although this Distancing is lower than that of insecure children. These data seem to reinforce the notion that attachment security enhances the development of more realistic and adequate mechanisms for evaluating reality (of oneself and others) [50,51], which in turn can promote more filial maturity.

In addition to their combined effect, attachment and mental representation of caregiving are also separately relevant for the development of filial maturity, but in a differentiated way according to each of its dimensions. That is, MRC is significant in explaining Comprehending and attachment in explaining Distancing. Thus, we can anticipate that the greater perception of ability and availability to care and the perception of others as deserving of care facilitate the development of the ability to establish intimate, understanding and supportive relationships (Comprehending). In other words, children would hardly be available for close, understanding and supportive filial relationships if they did not have these representations. On the other hand, children with secure attachment will have positive and flexible IWMs of themselves and the parents, which are essential for developing the ability to accept that parents also have weaknesses, flaws and limitations (Distancing). It is very likely that the representations of self and others and the ways of relating that children have developed in relationships with their parents, and that are active throughout their lives, influence the way in which, in adult life, they position themselves in the relationship with aging parents, specifically in the way they understand and interpret their potential and limitations and in the way they relate to them.

In addition, one of the factors that has been pointed out as a facilitator of filial maturity is openness and mutual knowledge between children and parents [15], which is closely associated with secure attachment. Likewise, negative emotions towards parents, resentments and unresolved conflicts are obstacles to the objectivity necessary for the development of mature relationships with parents, which is typical of insecure (avoidant and anxious) individuals.

In summary, considering the overall results of the regression analyses, we can consider that both attachment and MRC may constitute developmental precursors of filial maturity.

Still, some potential limitations of this study should be addressed. The sample is not representative, but its size is significant. Furthermore, the sociodemographic characteristics between men and women are different, which makes this sample specific and may limit the generalization of the results. Also, attachment and mental representation of caregiving were evaluated at a general level, whereas filial maturity refers specifically to the relationship with aging parents. The study shows that attachment and MRC moderately predict filial maturity, implying that other variables can be involved and added to the accounted variance. In the future, other factors should be analyzed, such as filial anxiety, the current engagement in regular parental caregiving activities, coping styles and personality characteristics. Lastly, all the variables were assessed through self-reporting. It is important that future research takes these issues into account.

## 5. Conclusions

In summary, the results show that younger adult children, who perceive themselves as more able and available to provide care and who evaluate others as deserving of help also have greater ability to establish relationships with parents based on intimacy, understanding and mutual support. Also, in secure attached children, the ability to see parents objectively, recognizing their abilities and needs as they are, increases when the ability and availability to provide care also increases, and the more they evaluate others as deserving of care.

This study contributes significantly to knowledge in such a complex and still poorly studied area as filial relationships in middle age and filial maturity, pointing out filial maturity and filial caregiving as developmental tasks of midlife, filial maturity as a facilitator of this task, and attachment and mental representation of caregiving as developmental resources that enhance adaptation to the challenges that the aging of parents poses within the scope of the filial relationship.

## Figures and Tables

**Table 1:** Sociodemographic characteristics.

	Male (*n* = 97)		Female (*n* = 207)		Total (*n* = 304)	
	*n*	%	*n*	%	*n*	%
Age (35–64)						
M (SD)	50.48 (7.75)		48.56 (7.79)		49.17 (7.82)	
Education						
M (SD)	11.51 (4.70)		13.22 (4.73)		12.67 (4.78)	
Marital status						
Single	14	14.40	33	15.90	47	15.50
Married/Living together	72	74.20	146	70.50	218	71.70
Divorced/Separated	10	10.30	23	11.10	33	10.90
Widowed	1	1.00	5	2.40	6	2.20
Professional status						
Employed	77	79.40	157	75.80	234	77.00
Full-time	62	63.90	137	66.20	199	65.60
Part-time	15	15.50	20	9.70	35	11.50
Unemployed	7	7.20	31	15.00	38	12.50
Retired	13	13.40	19	9.20	32	10.50
Children (0–4)						
M (SD)	1.40 (0.92)		1.36 (0.92)		1.37 (0.92)	
Brothers						
M (SD)	2.32 (2.22)		2.10 (2.02)		2.17 (2.08)	
Distance from parent						
M (SD)	19.87 (49.35)		17.90 (48.08)		18.54 (48.42)	
Parents’ functionality in BADL *						
Independent	62	63.90	136	65.70	198	65.10
Slightly dependent	17	17.50	42	20.30	59	19.40
Moderately dependent	4	4.10	11	5.30	15	4.90
Severely dependent	7	7.20	8	3.90	15	4.90
Totally dependent	7	7.20	10	4.80	17	5.60
Parents’ functionality in IADL *						
Independent	5	5.2	32	15.50	37	12.20
Moderately dependent	52	53.60	89	43.00	141	46.40
Severely dependent	40	41.20	86	41.50	126	41.40

* The parent with the highest degree of dependence was considered.

**Table 2:** Attachment style and type.

	Male (*n* = 97)		Female (*n* = 207)		Total (*n* = 304)		
	*n*	*%*	*n*	*%*	*n*	*%*	*χ* ^2^
Attachment style							0.05
Secure	54	55.7	118	57.0	172	56.6	
Anxious	35	36.1	72	34.8	107	35.2	
Avoidant	8	8.2	17	8.2	25	8.2	
Attachment type							0.05
Secure	54	55.7	118	57.0	172	56.6	
Insecure	43	44.3	89	43.0	132	43.4	

**Table 3:** Mental representation of caregiving and filial maturity.

	Male (*n* = 97)	Female (*n* = 207)	
	*M* (*SD*)	*M* (*SD*)	*t* (302)
**Mental representation of caregiving**			
MRC-1 ^a^	5.39 (0.60)	5.67 (0.57)	−3.80 ***
MRC-2 ^b^	4.83 (0.88)	5.48 (0.84)	−5.24 ***
MRC-3 ^c^	2.08 (0.69)	1.84 (0.56)	2.98 **
MRC-4 ^d^	4.97 (1.28)	5.17 (1.30)	−1.28
**Filial Maturity**			
Comprehending	3.49 (1.15)	3.90 (1.13)	−2.87 **
Distancing	3.54 (1.25)	3.79 (1.17)	−1.73

Note: ** *p <* 0.01; *** *p <* 0.001. Note: ^a^ perceived ability to provide effective help; ^b^ perceived ability to recognize others’ needs; ^c^ egoistic motives for helping; ^d^ appraisal of others as worthy of help.

**Table 4:** Change statistics for variables predicting Comprehending and Distancing.

Model	*R*	*R* ^2^	*Adjusted R* ^2^	Change Statistics				
				*R^2^ Change*	*F Change*	df1	df2	Sig. *F Change*
Comprehending ^†^								
1 ^a^	0.252	0.064	0.041	0.064	2.853	7	294	0.007
2 ^b^	0.254	0.065	0.039	0.001	0.331	1	293	0.566
3 ^c^	0.474	0.225	0.193	0.160	14.951	4	289	0.000
4 ^d^	0.496	0.246	0.212	0.021	8.202	1	288	0.004
Distancing								
1 ^a^	0.128	0.016	−0.007	0.016	0.704	7	296	0.669
2 ^b^	0.234	0.055	0.029	0.038	11.987	1	295	0.001
3 ^c^	0.252	0.064	0.025	0.009	0.693	4	291	0.597
4 ^e^	0.311	0.096	0.056	0.033	10.518	1	290	0.001
5 ^f^	0.333	0.111	0.068	0.014	4.607	1	289	0.033

^a^ Predictors: (constant), sociodemographic variables. ^b^ Predictors: (constant), sociodemographic variables, attachment. ^c^ Predictors: (constant), sociodemographic variables, attachment, MRC. ^d^ Predictors: (constant), sociodemographic variables, attachment, MRC, Attachment X RMPC-2. ^e^ Predictors: (constant), sociodemographic variables, attachment, MRC, Attachment X RMPC-1. ^f^ Predictors: (constant), sociodemographic variables, attachment, MRC, Attachment X RMPC-1, Attachment X RMPC-4. ^†^ 2 cases whose standardized residuals were below −3.00 were excluded.

**Table 5:** Summary of hierarchical multiple regression analysis for variables predicting Comprehending and Distancing.

Final Model	Unstandardized Coefficients		Standardized Coefficients		
	*B*	Standard Error	*β*	*t*	Sig.
Comprehending					
Gender	0.126	0.134	0.052	0.941	0.347
Age	−0.022	0.008	−0.155	−2.777	0.006
Education	−0.124	0.134	−0.053	−0.922	0.357
Marital status	−0.079	0.160	−0.031	−0.496	0.621
Professional status	−0.154	0.156	−0.057	−0.987	0.325
Children	−0.016	0.178	−0.006	−0.090	0.928
Siblings	0.120	0.171	0.037	0.703	0.483
Attachment	−2.034	0.714	−0.889	−2.850	0.005
MRC-1	0.640	0.105	0.334	6.097	0.000
MRC-2	−0.197	0.097	−0.154	−2.041	0.042
MRC-3	−0.149	0.106	−0.080	−1.403	0.162
MRC-4	0.147	0.047	0.168	3.114	0.002
Attachment X MRC-2	0.387	0.135	0.919	2.864	0.004
Distancing					
Gender	0.219	0.157	0.085	1.396	0.164
Age	−0.007	0.009	−0.044	−0.723	0.470
Education	0.024	0.153	0.010	0.157	0.875
Marital status	0.102	0.182	0.038	0.560	0.576
Professional status	−0.040	0.179	−0.014	−0.222	0.824
Children	−0.113	0.202	−0.038	−0.560	0.576
Siblings	0.011	0.197	0.003	0.056	0.955
Attachment	−5.615	1.363	−2.320	−4.119	0.000
MRC-1	−0.349	0.177	−0.173	−1.969	0.050
MRC-2	−0.131	0.084	−0.097	−1.559	0.120
MRC-3	−0.051	0.122	−0.026	−0.423	0.673
MRC-4	−0.076	0.080	−0.082	−0.946	0.345
Attachment X MRC-1	0.713	0.230	1.679	3.098	0.002
Attachment X MRC-4	0.229	0.107	0.517	2.146	0.033

## Data Availability

The raw data supporting the conclusions of this article will be made available by the authors upon request. The data are not publicly available due to privacy or ethical restrictions.
